# Using Seabird Habitat Modeling to Inform Marine Spatial Planning in Central California’s National Marine Sanctuaries

**DOI:** 10.1371/journal.pone.0071406

**Published:** 2013-08-13

**Authors:** Jennifer McGowan, Ellen Hines, Meredith Elliott, Julie Howar, Andrea Dransfield, Nadav Nur, Jaime Jahncke

**Affiliations:** 1 Department of Geography, San Francisco State University, San Francisco, California, United States of America; 2 Point Blue Conservation Science, Petaluma, California, United States of America; Hawaii Pacific University, United States of America

## Abstract

Understanding seabird habitat preferences is critical to future wildlife conservation and threat mitigation in California. The objective of this study was to investigate drivers of seabird habitat selection within the Gulf of the Farallones and Cordell Bank National Marine Sanctuaries to identify areas for targeted conservation planning. We used seabird abundance data collected by the Applied California Current Ecosystem Studies Program (ACCESS) from 2004–2011. We used zero-inflated negative binomial regression to model species abundance and distribution as a function of near surface ocean water properties, distances to geographic features and oceanographic climate indices to identify patterns in foraging habitat selection. We evaluated seasonal, inter-annual and species-specific variability of at-sea distributions for the five most abundant seabirds nesting on the Farallon Islands: western gull (*Larus occidentalis*), common murre *(Uria aalge),* Cassin’s auklet (*Ptychorampus aleuticus*), rhinoceros auklet (*Cerorhinca monocerata*) and Brandt’s cormorant (*Phalacrocorax penicillatus*). The waters in the vicinity of Cordell Bank and the continental shelf east of the Farallon Islands emerged as persistent and highly selected foraging areas across all species. Further, we conducted a spatial prioritization exercise to optimize seabird conservation areas with and without considering impacts of current human activities. We explored three conservation scenarios where 10, 30 and 50 percent of highly selected, species-specific foraging areas would be conserved. We compared and contrasted results in relation to existing marine protected areas (MPAs) and the future alternative energy footprint identified by the California Ocean Uses Atlas. Our results show that the majority of highly selected seabird habitat lies outside of state MPAs where threats from shipping, oil spills, and offshore energy development remain. This analysis accentuates the need for innovative marine spatial planning efforts and provides a foundation on which to build more comprehensive zoning and management in California’s National Marine Sanctuaries.

## Introduction

The past decade has seen substantial growth in policy and framework development on how to better manage oceans through marine spatial planning; an approach that integrates ecosystem science, human activities, stakeholder consensus and conservation objectives to improve ocean governance [1]. There is a growing foundation of work on ecosystem-based management [2], tools for planners with decision-making power [3], [4] and strategies that quantify the impacts of human activities on marine and coastal resources [5], [6], [7]. Several states in the United States (U.S.), such as California, Oregon and Massachusetts among others, continue to coordinate with policymakers, scientists and stakeholders to incorporate marine spatial planning into the development and management of comprehensive zoning plans in their jurisdictional waters [7], [8]. In 2010, President Obama issued Executive Order 13547 and formally brought marine spatial planning into the nation’s coastal and marine resource management program [9]. While this process is underway, comprehensive zoning does not exist for Federal waters, which include National Marine Sanctuaries.

Sanctuaries are distinguished areas of ecological or cultural importance with well-defined boundaries, making them ideal spatial units for zoning and prioritization exercises. As with most marine environments, Sanctuaries face persistent pressure from commercially and recreationally valued human activities (industrial shipping, commercial fishing, recreational and military activities, etc) that impact biodiversity in different ways [10]. Managers adaptively regulate these activities and re-evaluate management plans approximately every five years to ensure the coordinated preservation of Sanctuary resources [11]. Studies that can assist Sanctuary scientists and managers in making informed decisions regarding threat mitigation and implementation of ecosystem-level management are considered a top priority [10].

West coast Sanctuary managers oversee offshore areas situated in the productive California Current System (CCS). This eastern boundary current spans the western edge of North America from British Colombia to Baja, California [12]. Strong upwelling in this system results in increased productivity that enhances foraging opportunities for vast numbers of marine mammals, seabirds, and commercial fish throughout the year [13], [14], [15]. The Gulf of the Farallones (GFNMS, est. 1981) and Cordell Bank (CBNMS, est. 1989) National Marine Sanctuaries provide habitat and breeding ground for over a half a million seabirds including several endangered species such as the Ashy-storm petrel [16]. The Farallon Islands, located 45-km west of San Francisco Bay, are comprised of a cluster of granite outcroppings that support the largest breeding seabird colonies in the continental U.S. [16]. Cordell Bank, a 7.2-km by 15.2-km underwater seamount at the edge of the continental shelf, supplies a biodiverse benthic ecosystem with nutrient-rich offshore waters [10]. Gaining biophysical and spatial understanding of species’ distributions and habitat use within Sanctuary waters will facilitate the prioritization of current and future conservation efforts [10].

Here, we propose using modeled seabird habitat to inform spatial planning at the Sanctuary level. Seabirds are particularly sensitive to changes in food supply driven by shifts in ocean climate regimes [17], [18], [19]. This supports their use as bio-monitors or indicator species of marine food web dynamics and ocean health [20], [21], [22]. Their conspicuousness and colonial nature makes the acquisition of large amounts of data possible at or near predictable breeding site locations. This is advantageous to conservation managers, in that, at-sea distributions of seabirds can be used to identify important areas for more elusive or data-deficient marine species [23], [24]. Investigating seabird distributions within larger systems is a growing focus of research that has the potential to inform marine protected area planning, locate important areas for migratory species, and contribute to a better understanding of localized ecosystem structuring [22], [25], [26], [27], [28].

The factors driving at-sea habitat selection often remain poorly understood due to marine species exploiting dynamic oceanic phenomenon that drive prey availability [29], [30], [31] and that change in space and time [32]. Within the CCS, distributions have been delineated through modeling associations between seabirds and bathymetric and oceanographic features [13], [14], [15] surface nekton [33] and krill [34]. Nur et al. [24] modeled the predictive abundance of 16 individual species and aggregated them to identify persistently used foraging habitat at the system-wide scale of the CCS. Highly variable species responses to ecological phenomenon in the CCS mean multi-species and multi-scalar studies are required to enhance our understanding of at-sea distributions for management purposes [22]. Our study contributes to this body of knowledge by modeling the finer-scale (≤3-km) habitat associations of individual seabird species within Sanctuary boundaries to identify high use foraging areas, as these areas are locally significant and should be supported by Sanctuary-level conservation efforts [24], [28].

There are many tools available to help facilitate spatial prioritization of ocean waters by identifying spaces for conservation, recreation, and human uses within planning networks. Site selection algorithms, such as Marxan (The University of Queensland website, Available: http://www.uq.edu.au/marxan/marxan-software. Accessed 2013 Jul 13) quantitatively address user-defined spatial questions, and have already been used in several studies to aid marine spatial planning in California [35], [36], [37]. In this study, we use Marxan to inform future ocean zoning and improve marine conservation within Sanctuary boundaries. Our objectives are to: 1) identify the primary drivers of localized foraging habitat selection for the resident breeding seabird species of the Farallon Islands based on near surface water properties, bathymetric features, and oceanographic climate regimes; 2) predict seabird abundance and distribution patterns per and across species to determine highly selected foraging areas within the Sanctuaries; and 3) conduct a spatial prioritization exercise using Marxan to examine potential seabird conservation areas where human-wildlife conflicts would be minimized. This paper serves as a pragmatic example of how species distribution modeling and innovative conservation planning tools can be used to develop comprehensive zoning at scales useful for local management.

## Materials and Methods

### Study Area and Survey Design

**Figure 1:**
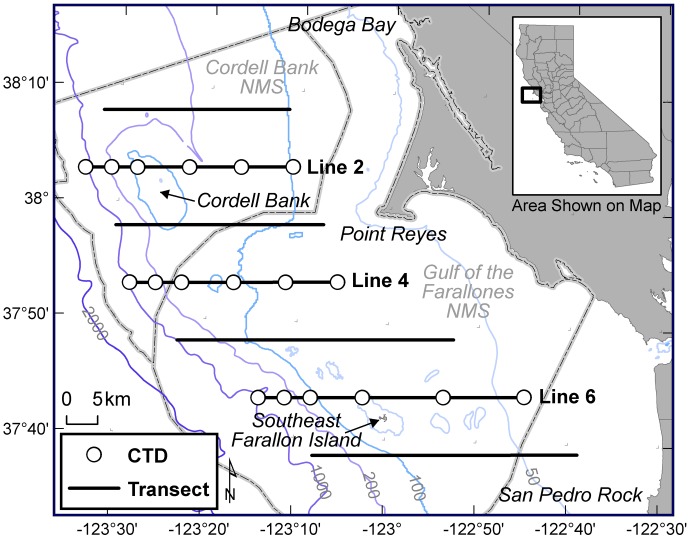
Location of the study area in California and primary ACCESS program transects with designated CTD sampling stations (Jahncke et al. 2008).

We used survey data collected over 8 years by the Applied California Current Ecosystem Studies (ACCESS) program, conducted by Point Blue Conservation Science (formerly Point Reyes Bird Observatory), Gulf of the Farallones NMS and Cordell Bank NMS (ACCESS website. Available:www.accessoceans.org. Accessed 2013 Jun 7). The Sanctuaries are located in north central California and span 851 km^2^ and 2063 km^2^, respectively. ACCESS implements a yearly sampling scheme designed to monitor marine birds and mammals, surface and water column properties and zooplankton abundance within the Sanctuaries. The 27 data cruises included in this study span from April through September from 2004 through 2011. ACCESS transects ran east-west and spanned the continental shelf and slope defined by the 50-m to the 1000-m isobaths to cover the offshore area between southern Bodega Bay (38° 8′ N) and San Pedro Rock (37° 21′ N: Fig. 1).

The number of transects covered during each cruise varied as a function of time and weather conditions. Figure 1 shows the primary transects used in this analysis (1–7), as these are the most frequently surveyed and encompass the spatial extent of oceanographic and water-column sampling stations. Previous studies investigating spatial autocorrelation for this dataset concluded 3-km line segments, referred to as bins, to be an appropriate size [14], [24]. We followed these methods and binned seabird data along transect lines, represented by a single midpoint (Fig. 1). For every data bin, we integrated corresponding information on transect specifications and survey environment (Table 1). Smaller bin sizes occasionally arose at the end segments of transect lines; bin sizes <1-km were discarded from the analysis.

**Table 1:** Description and ranges of habitat-specific variables used to model seabird abundance at 3-km bins.

Variable	Description	Mean±SD	min-max values	CV
***Oceanographic***				
SST (°C)	Average surface temperature for 3-km bin	12.6±1.62	8.9–16	0.13
SSS (psu)	Average surface salinity for 3-km bin	33.3±0.48	29.7–34	0.02
SSF (mg/m^3^ )	Average surface fluorescence for 3-km bin	1.08±2.23	0–14.9	2.06
***Bathymetric***				
Dist_Land (m)	Distance from bin midpoint to mainland	27016±9396	1210–47790	0.34
Dist_200 (m)	Distance from bin midpoint to 200m-isobath	10759±9315	23–47260	0.86
Dist_SEFI (m)	Distance from bin midpoint to South East Farallon Island	29062±17211	1273–65366	0.59
***Climate Indices***				
NPGO	Monthly North Pacific Gyre Oscillation value	0.51±0.94	**−**1.4–1.9	
PDO	Monthly Pacific Decadal Oscillation value	**−**0.1±1.01	**−**1.8–1.86	
SOI	Monthly Southern Oscillation Index value	0.41±1.76	**−**2.7–4.3	
UI value	Ten-day average to last day on monthly cruise	93.0±40.2	6.4–171.5	0.43
***Detection Biases***				
Strip width (m)	Observer field of vision per 3-km bin	167.8±79.2	50–300	0.47
Sea State	Observed Beaufort scale conditions	2.56±1.28	0–6	0.50
Swell Height (m)	Observed swell height per 3-kmbin	1.98±5.07	0–8	5.91
Visibility	Observer visibility per 3-km bin	5.51±2.04	0–9	0.38
Time of Day	Hour:Min of survey bin completion	1217±0304	0608–2005	0.24
Cloud Cover	Observed values recorded per 3-km bin	5.21±3.37	0–9	0.65

### Species Data

Seabird surveys were conducted from the flying bridge of three research vessels (R/V) of varying size: the smallest R/V John Martin, medium R/V National Oceanic and Atmospheric Administration (NOAA) Fulmar, and largest R/V McArthur II. Surveys used standardized strip-survey methods [38] to continuously count birds off the side of the vessel with the best visibility (lowest glare) and during daylight hours while the vessel was underway at 10 knots [18]. Seabirds encountered within a 90°arc from the bow to the survey side of the vessel, and within a range of 50–300-m, were counted by a single observer (see Jahncke et al. 2008 for further details) [18]. Variable strip width was dependent on the vessel used: Martin (∼100-m), Fulmar (∼200-m) and McArthur II (∼300-m). Strip-widths of <100-m reflect field survey adjustments for situations when poor visibility limited the surveyor’s range of vision. Seabird behaviors were recorded (flying or commuting, foraging, ship attract, and sitting on the surface of the water) and only records of birds foraging, feeding or sitting on the sea surface were used in the analysis and assumed to be either actively foraging or resting post prey consumption [39].

Surveys encompassed the seabird breeding season when adult movement between the main colony on Southeast Farallon Island and foraging areas is limited by energy demands [40], as well as pre- and post- breeding months. While there are 13 seabird species breeding on the Farallon Islands, this study focuses only on the most abundant species recorded throughout the temporal span of surveys (>100 survey-bins with observations; Table 2). The five focal species are: western gull (*Larus occidentalis*), common murre *(Uria aalge*), Cassin’s auklet (*Ptychorampus aleuticus*), rhinoceros auklet (*Cerorhinca monocerata*), and Brandt’s cormorant (*Phalacrocorax penicillatus)*. The total number of seabirds counted along transect lines were totaled by species at each 3-km bin and recorded to the corresponding midpoint.

**Table 2:** Sample sizes for focal species noting the number of zero and non-zero bins and the maximum individuals sighted within a bin (n = 2336).

Acronym	Species common name	Zero-count bins	Non-zero counts bins	Count maximum
WEGU	Western gull	1918	418	68
COMU	Common murre	1172	1164	1216
CAAU	Cassin’s auklet	1809	527	789
RHAU	Rhinoceros auklet	1995	341	45
BRAC	Brandt’s cormorant	2158	178	300

### Environmental Variables

Oceanographic Data. Sea surface characteristics were recorded *in situ* by a thermosalinograph installed in the sea chest of each ship. Sea surface temperature (SST), salinity (SSS) and fluorescence (SSF) values were processed and averaged to the corresponding transect bins matching seabird observations. On 11 cruises within the dataset, the vessels were not equipped with a thermosalinograph in the hull and did not continuously collect SSF data. However, all cruises, regardless of vessel, recorded fluorescence using a Sea-Bird Electronics SBE 19*Plus* SEACAT Conductivity-Temperature-Depth (CTD) Profiler equipped with a WetStar Fluorometer at 15–18 designated CTD sampling stations designed to continuously sample oceanographic water-column properties from the surface to the seafloor (Figure 1). We averaged CTD fluorescence values collected within the 1-6-m depth range at these stations to assign surface values at each station per cruise for a total of 740 records. We then linearly regressed these values, along with year and SST to predict missing SSF records at the 3-km bin locations for the 11 cruises. Any records missing SST or SSS values were not included in the analysis.

Distance Measurements. We calculated distances in ArcGIS (10.0, ESRI Redlands, CA) from bin midpoints to the nearest oceanic shelf break at the 200-m isobath, derived from the California Department of Fish and Wildlife 200-m EEZ bathy-topo grid (DFG website. Available: http://www.dfg.ca.gov/marine/gis/downloads.asp. Accessed 2013 Jul 13). Sanctuary and California boundary shapefiles were accessed online through the National Marine Sanctuary Geographic Information System Dataset (NOAA website. Available: http://www.sanctuaries.noaa.gov-/library/imast_gis.html. Accessed 2013 Jun 6). We also used these shapefiles to calculate midpoint distances to nearest coastal land and to the Southeast Farallon Island as it supports the largest breeding colonies of each species in the Sanctuaries.

Climate Indices. Seasonal and inter-annual variability in oceanographic trends directly influence the amount of upwelling that occurs in the CCS [12], [18]. We included several ocean climate indices known to drive variability in the CCS [24]; [41]: 1) the North Pacific Gyre Oscillation (NPGO) exhibits correlations with salinity and productivity as depicted by chlorophyll-a (chl-a) along the western coast of North America [42]; 2) the Pacific Decadal Oscillation (PDO) is the primary driver of North Pacific SST poleward of 20°N [43]; and 3) the Southern Oscillation Index (SOI) records the warming and cooling trends of the tropical Pacific ocean in relation to El Niño and La Niña events [44]. Monthly values for each oceanic climate index were assigned to each bin midpoint based on cruise year and month, as were daily upwelling values collated by the Pacific Fisheries Environmental Laboratory (PFEL website. Available:http://www.pfeg.noaa.gov/products/PFEL/modeled/indices/PFELindices.html. Accessed 2013 Jul 13) to account for local upwelling conditions prior to each cruise (10-day average leading up to the last day of the cruise) [45].

### Human Uses for Spatial Prioritization

Human use layers were created by a partnership between NOAA’s Marine Protected Areas Center and the Marine Conservation Institute through a series of statewide participatory workshops, and released to the public as the California Ocean Uses Atlas (NOAA website. Available: http://www.mpa.gov/dataanalysis/atlas_ca/. Accessed 2013 Jul 13). These maps document 30 human activities occurring within California’s exclusive economic zone. Data came in the form of polygons created in a GIS that outline the spatial extent of each activity in coastal waters as delimited by stakeholder knowledge within distinct regions. Each activity contained multiple sets of polygons describing regional stakeholder use under general (used with some regularity), dominant (targeted by most users), and future (expansion/intensifying of use) footprints of activities at the time of issue in 2010.

### Data Analysis and Model Fitting

Species distribution modeling is a growing discipline in both theory [46], [47], [48] and application [49], [50], [51], [52]. Methods require the extrapolation of a fitted model in a GIS to produce a series of predictive surfaces within a spatially explicit framework [53]. There are substantial reviews of distribution modeling methods, accompanying uncertainties and recommendations for ecological modeling improvements [48], [54], [55], [56], [57], [58]. While species distribution models, like any abstracted modeling approach, are limited by their tendencies towards over-simplification of complex biophysical and ecological interactions, they provide helpful insight and perform well as predictive tools when modeling species distributions within the range used in this analysis [58]. Of available modeling tools, Generalized Linear Models (GLMs) prove to be a statistically strong approach used in conservation and biodiversity studies.

**Figure 2:**
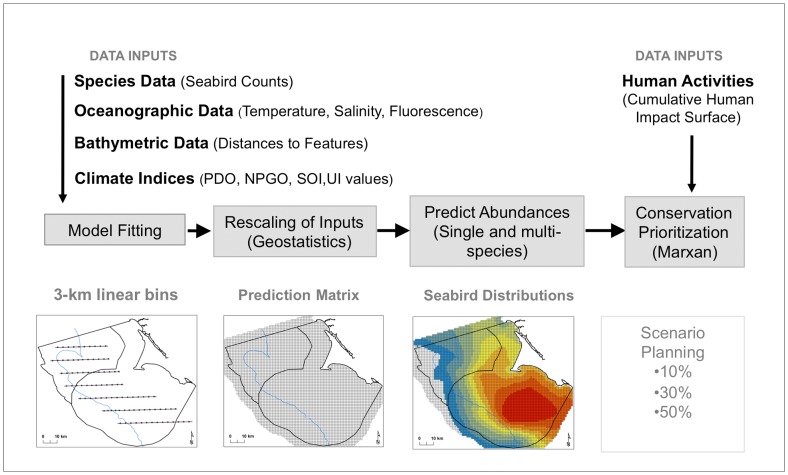
Flow chart of methodology adapted from Franklin (2009).

Our methodological design drew from Franklin [50] in order to encompass both ecological reasoning and predictive spatial tools when modeling for management purposes. A conceptualized model of our process is provided in Figure 2. Treating seabird counts as our dependent variables, we first used negative binomial regression to test all covariates for linear or quadratic relationships while controlling for month and year in Stata 10 (StataCorps, 2007, Stata Statistical Software, College Station, TX: StataCorp LP). After testing for multicollinearity between variables and finding no high correlations (VIF <10) [59], all potential covariates were added to a preliminary negative binomial model and eliminated using manual backward stepwise negative binomial regression for model simplification at the 0.05 significance level. Because we were modeling counts and not densities, we used an exposure coefficient in all models to account for varying bin sizes and strip widths (area range: 0.05–0.9). For the exposure coefficient we used the log of the binned areas to incorporate differences in rates of detection across surveyed bins.

Since our 2,336 observation dataset contained large numbers of zeros (Table 2), we used a zero-inflated methodology to model abundance. Zero-inflation is a distinct characteristic of field-collected count data, in that, excess zeros arise from observer detection biases that may fail to reflect suitable habitat in the absence of a species. Ignoring excess zeros may result in models with problematic inferences and incorrect assumptions of ecological linkages between species and environment [60]. Zero-inflated models can provide better fits when dealing with excess zeros as they account for both true (unsuitable habitat) and false zero observations [61]. Although survey-strip transect methods traditionally assume perfect detectability within a given strip width [38], false zero records can occur in the marine environment from a variety of ecological and environmental circumstances. We refer to factors influencing zero-inflation as detection bias variables. For example, a species may not be observed because sea conditions obscure visibility at the time a survey passes [62]. In recent years, there has been considerable interest directed at modeling excess zeros for ecological applications and predictive mapping that informs policy and management decisions (see Zuur et al. 2009 for more for details) [61], [63], [64], [65].

All significant variables retained from the preliminary model were added to a zero-inflated negative binomial model that also included detection bias variables. The bias variables used in this study came from assessments of environmental conditions recorded by scientific observers during the surveys, and time, which was analyzed as a six-digit numeric value, hhmmss (Table 1). If detection bias variables maintained a significance level of 0.05 they were included in the model. For all parameters, we then used a manual backward stepwise procedure for overall model simplification to allow for biological interpretation instead of automated model reduction [46], [66]. We used two test statistics: the log-likelihood of alpha to determine the level of significance for models using negative binomial over Poisson regression, and the Vuong test statistic to determine model preference for zero-inflated negative binomial regression over standard negative binomial regression [67]. Because some of the years in our study period included anomalous ocean conditions, we tested for interactions between year (as a categorical variable) and significant oceanographic variables (SST, SSS). Using log-likelihood ratio statistics, we compared nested models to identify the best-fit interaction. If models had more than one significant oceanographic variable and both interactions were significant, we selected the model with the strongest interaction. We used *n*-fold cross-validation to assess model fit by randomly splitting binned observations into mutually exclusive subsets [68]. We created multiple models (n = 10) for each species and cross-validated leaving one subset out each time.

### Predicting Species Distributions

To apply our findings to the larger Sanctuary area, we created surface water maps based on binned midpoint values of SST, SSS, and SSF using ordinary kriging as an interpolation method [33], [69]. Our interpolated values were predictions of oceanographic characteristics at unsampled locations, and in this study these values extended up to 25-km beyond survey coverage (Fig. 2). We interpolated surface profiles for each oceanographic variable per cruise using kriging because it allows for irregularities in non-parametric surfaces by quantifying their spatial structure and is flexible as both a local and global interpolator [69]. Since we were working within an eight-year temporal frame, and modeling three oceanographic surface profiles (SST, SSS, SSF), we were interested in capturing general trends across each of the 81 surfaces (3 variables×27 cruises). To accomplish this we employed parameter optimization for each variable on a cruise-by-cruise basis to automate the interpolation process. Optimization minimizes the mean square error for predictions by assuming data are isotropic or have no directional influences, and applies a default search radius to weight measured locations [69]. We plotted each variable based on their overall mean square error of predictions to identify outlying cruises, which we then further investigated for trends in the semivariogram and detrended when necessary to minimize errors across all months used in the analysis (Root Mean Squared Errors (mean ± SD): SST = 0.22±0.03; SSS = 0.07±0.05; SSF = 0.21±0.15). We kept high variability from anomalous weather events in the analysis as these occur naturally and are important to consider when modeling yearly interactions of species reactions to environmental changes [18], [41].

We then created a 1-km^2^ prediction cell data matrix set to the extent of the Sanctuaries with an additional 5-km buffer beyond Sanctuary boundaries to incorporate waters bordering our study area (Figure 2). We sampled the kriged surfaces on a cruise-by-cruise basis to extract SST, SSS, SSF information; calculated distances to cell centroids; and assigned the same monthly climate index values used in the model fitting. To complete the prediction data matrix we calculated the most frequently recorded values of detection bias variables to assign environmental classifications. We then modeled predicted abundances for each species per cruise at the scale of a single grid cell.

We followed the methodology used in Nur et al. [24] to identify highly used habitat on the basis of modeled abundance values derived from count data. We standardized abundance first across months and then across months and years based on the prediction cell average (1-km^2^) for each species. These values identified high-use habitat throughout the breeding season for both individual species and multi-species distribution maps. For individual species, we focused on the most consistent ACCESS cruise months: May, July and September. For multi-species models, standardization was completed prior to combining species to ensure equal contributions from each. Not addressing this would have biased habitat selection map results towards species with higher mean abundances. We repeated the standardization of species-specific abundances across months and years to produce a single, multi-species abundance value for each prediction cell to identify highly selected foraging areas within the Sanctuaries. For visualization purposes, we then ordered both single species and multi-species standardized abundances using percent ranking with the objective of mapping the level of habitat use in a particular cell [24].

### Spatial Prioritization

We consulted with Sanctuary scientists and managers to consolidate California Ocean Uses Atlas layers into those activities occurring inside Sanctuary boundaries that also demonstrated a varying degree of spatial coverage. We used polygon layers categorizing the dominant spatial coverage of each activity to highlight core areas within the Sanctuaries used for recreational, military, or socio-economic purposes. We did not include routine activities within Sanctuary waters for which we did not have spatially explicit information due to their ubiquity at the resolution of the data, i.e: recreational and pelagic commercial fishing.

Four regional experts (Sanctuary staff and scientists) discussed and scored the remaining activities from 1 (low) to 5 (high) using knowledge on the frequency of the activities, their passive disturbance to seabirds and potential for direct endangerment (i.e. the potential for an oil spill). The activities, scores and rationale are as follows: Military uses (disturbance:low; endangerment:very low) = 1; wildlife viewing (disturbance:low; endangerment:low) = 2; commercial benthic fishing with mobile gear (disturbance:low; endangerment:low) = 2; commercial benthic fishing with fixed gear (disturbace:low; endangerment:medium) = 3, industrial shipping(disturbance:medium; endangerment:high) = 4; and oil/gas shipping (disturbance: medium; endangerment: very high) = 5. Final scores were determined by group consensus for each activity and then applied to grid cells overlapping the spatial extent of the activity’s footprint. The sum of activities occurring in each grid cell then generated a general human use layer for incorporation into the spatial prioritization exercise (maximum value = 17).

Our strategy used the spatial prioritization tool Marxan to meet seabird conservation targets under different sets of constraints [70]. Marxan operates on the minimum-set problem, which can be described as the algorithm’s objective to satisfy the minimum target amount of an identified conservation feature for the lowest cost [71], [72]. Here, we used two scenarios to examine varying spatial solutions that met targets of 10%, 30% and 50% seabird habitat conservation with all other parameters held constant for both analyses. We designed scenario 1 to identify preferred areas for conservation from the perspective of the resource (seabird habitat) alone. To do so we used the species-specific modeled abundances (across months and years) as our conservation features and minimized area as a means to minimize cost. Scenario 2 identified preferred areas for conservation while simultaneously considering disturbance-causing activities from the general human use layer. We used the species penalty factor, a Marxan parameter that individually scales conservation features based on the importance of meeting their targets in solution outputs. This helped us incentivize meeting the targets of less abundant species such as Brandt’s cormorants and rhinoceros auklets, without compromising the more abundant ones (see Table 1). The targets reflect a spectrum of low to high-hypothesized conservation goals used in previous studies [35], [73]. Each scenario’s solutions were obtained from 100 iterations for each conservation target.

## Results

We used zero-inflated negative binomial regression to significantly model all species (Table 1), excluding common murres where the preferred model was standard negative binomial regression due to generally higher abundances and fewer zero counts (Table 1). Cassin’s auklets had the least significant of zero-inflated models with p = 0.06; however, controlling for potential bias was critically important for this small, hard to detect seabird. From all detection bias variables tested, only cloud cover, sea state and time significantly contributed to zero-inflation. Cloud cover influenced the detection of both auklet species. Sea state influenced the detection of rhinoceros auklets and western gulls. Time of surveys influenced detections of Cassin’s auklets and Brandt’s cormorants. Cross-validation results showed all models significantly predicted species abundance at a 0.05 level (Table 3).

**Table 3:** Species model results showing the best transformation (L = linear; Q = quadratic) and the sign of the coefficient for significant habitat variables, bolded oceanographic variables included interactions with year (n = 2336).

Variable	western gull	common murre	Cassin’s auklet	Rhinoceros auklet	Brandt’s cormorant
***Model Fitting***					
SST		**Q (−)** **			**Q(−)** *
SSS			**L (+)** ***	L (+)*	
SSF		Q (+)**	Q (+)*	L (**−**)**	
Dist_land		Q (**−**)***			
Dist_200m	Q(**−**)***	L (+)***	Q (**−**)***	L (**−**)***	L (+)***
Dist_SEFI	L (**−**)**	Q (+)***	Q(+)***	L (+)*	L (**−**)***
NPGO		Q (+)***		L (**−**)***	
PDO		Q (+)***			
SOI			Q (+)***	L (+)***	
UI_value	L (**−**)*				
Cloud			L (**−**)*	L (**−**)**	
Sea state	L (+)**			L (+)**	
Time of day			L (+)*		L (**−**)**
Model X^2^ (df)	70.7 (13)	1017.4(29)	397.8 (25)	311.5 (15)	336.9(20)
Model P	<0.0001	<0.0001	<0.0001	<0.0001	<0.0001
Vuong test for zero inflation	0.02		0.06	0.00	0.04
***Model Validation***					
Model F (1, 2334)	5.52	20.18	35.37	107.22	4.52
Model P	0.0189	<0.0001	<0.0001	<0.0001	<0.0336

* = p-value <0.05

** = p-value <0.01

*** = p-value <0.0001.

### Habitat Associations

We summarize seabird habitat associations as follows (Table 3): SSS and SSF significantly influenced distributions of two alcid species: Cassin’s auklets and rhinoceros auklets, yet showed no statistically significant relationship to the distribution of western gulls, common murres, or Brandt’s cormorants. SST was a significant habitat driver for common murres and Brandt’s cormorants. In addition, the influence of oceanography on the number of individuals observed varied among years as evidenced by the inclusion of significant year-interaction terms for three species: common murres, Cassin’s auklets, and Brandt’s cormorants. Oceanographic variables did not contribute to the western gull distribution model. Bathymetric features, namely distance to SEFI and the shelf break were significant predictive variables for all species models. Climate indices significantly influenced the alcid species with NPGO and PDO contributing to common murre distributions, SOI contributing to both auklet distributions and PDO influencing rhinoceros auklets. Western gulls showed no relationship to these Pacific-basin scale indices, but instead exhibited significant correlations to coastal upwelling.

### Predicted Distributions

**Figure 3:**
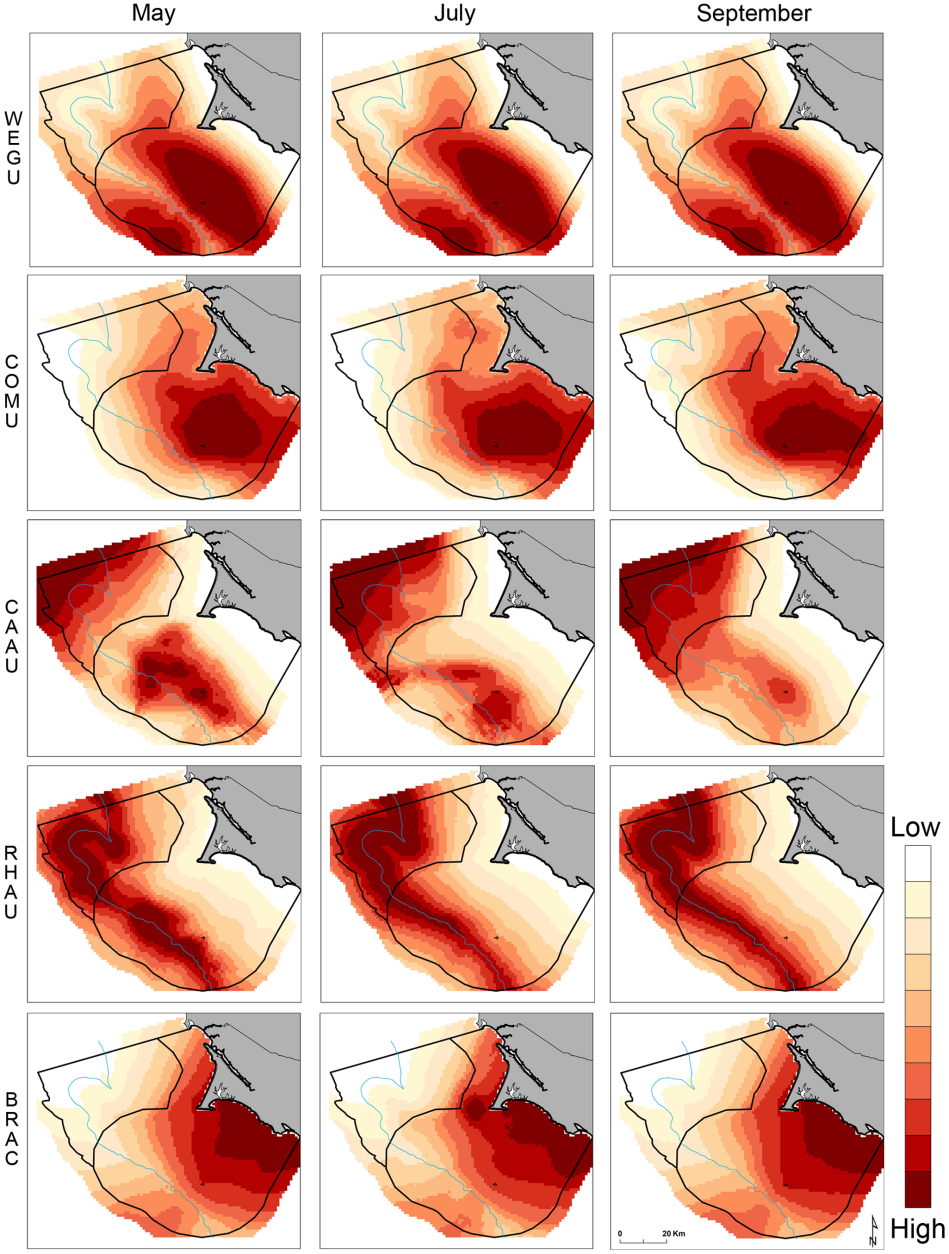
Species-specific variations in modeled habitat use for May, July, and September months (2004–2011); each gradient represents a 10% difference in habitat selection.

**Figure 4:**
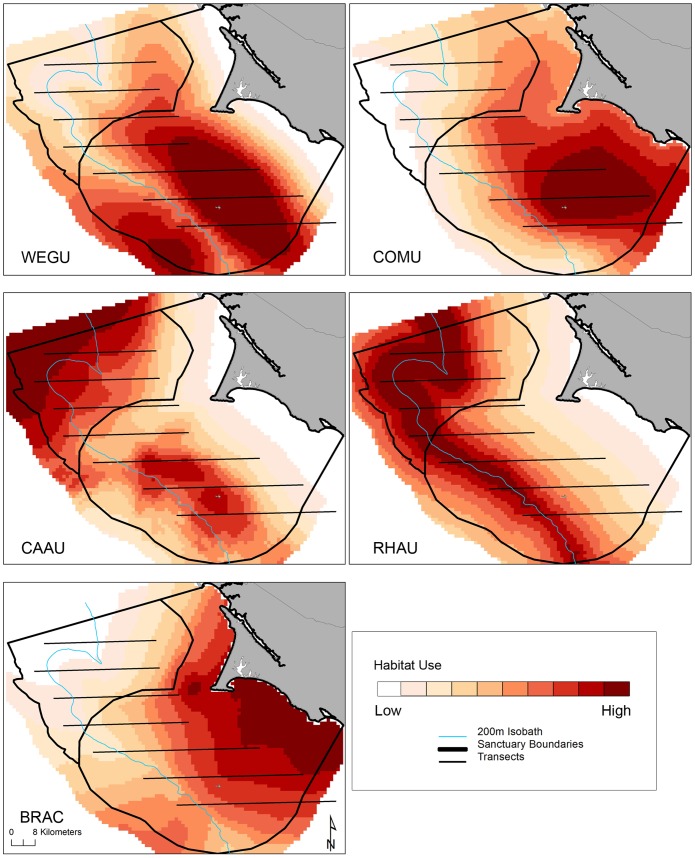
Species-specific habitat use across months and years (2004–2011); each gradient represents a 10% difference in modeled habitat selection.

**Figure 5:**
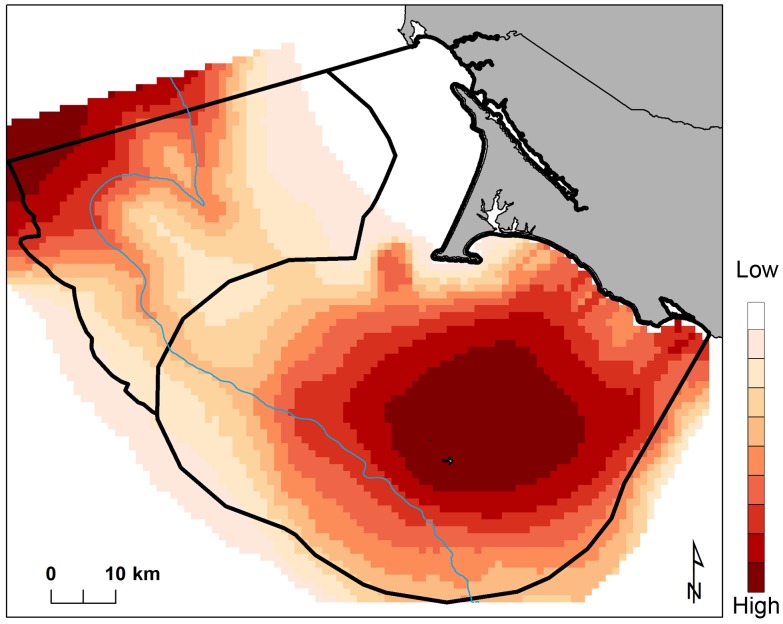
Multi-species high-use foraging areas across months and years (2004–2011); each gradient represents a 10% difference in modeled habitat selection.

Differences in habitat selection varied slightly across months for species whose distributions were driven by oceanographic variables (Figure 3). Distance to static bathymetric features were the most significant driver of western gull distributions and therefore no spatial variation across months can be seen. Figure 4 captures distinct distributions generated from standardized modeled abundances, across all months and years, to reflect species-specific habitat use throughout the eight-year data range. Here, transects delimit the spatial coverage of survey lines to highlight areas where prediction artifacts are likely present (see Discussion). The composite multi-species map highlights two areas used by all species: the waters northwest of Cordell Bank, and the waters surrounding the Farallon Islands with eastward, asymmetric directionality moving along the shelf towards the mainland (Figure 5).

### Spatial Prioritization

**Figure 6:**
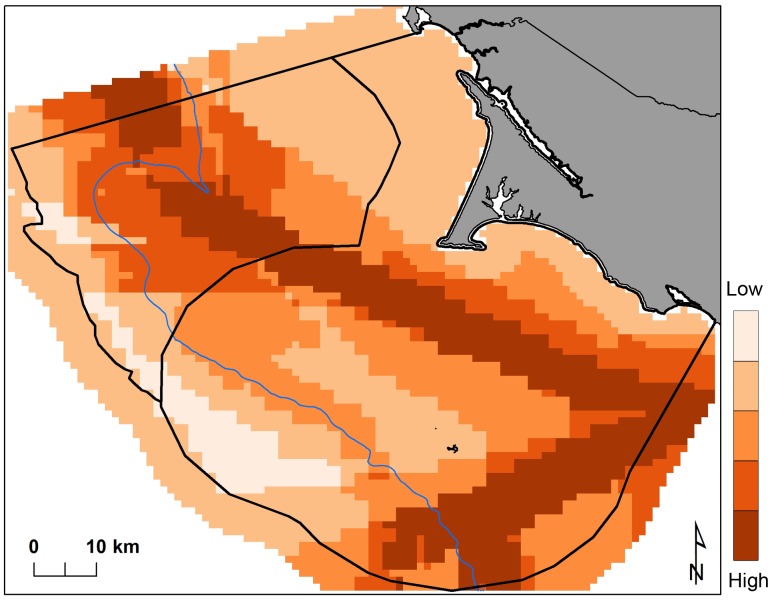
Human uses based on the sum of impact scores and the distribution of all activities occurring per 1 km^2^ cell; each gradient represents a 20% increase in impact score.

**Figure 7:**
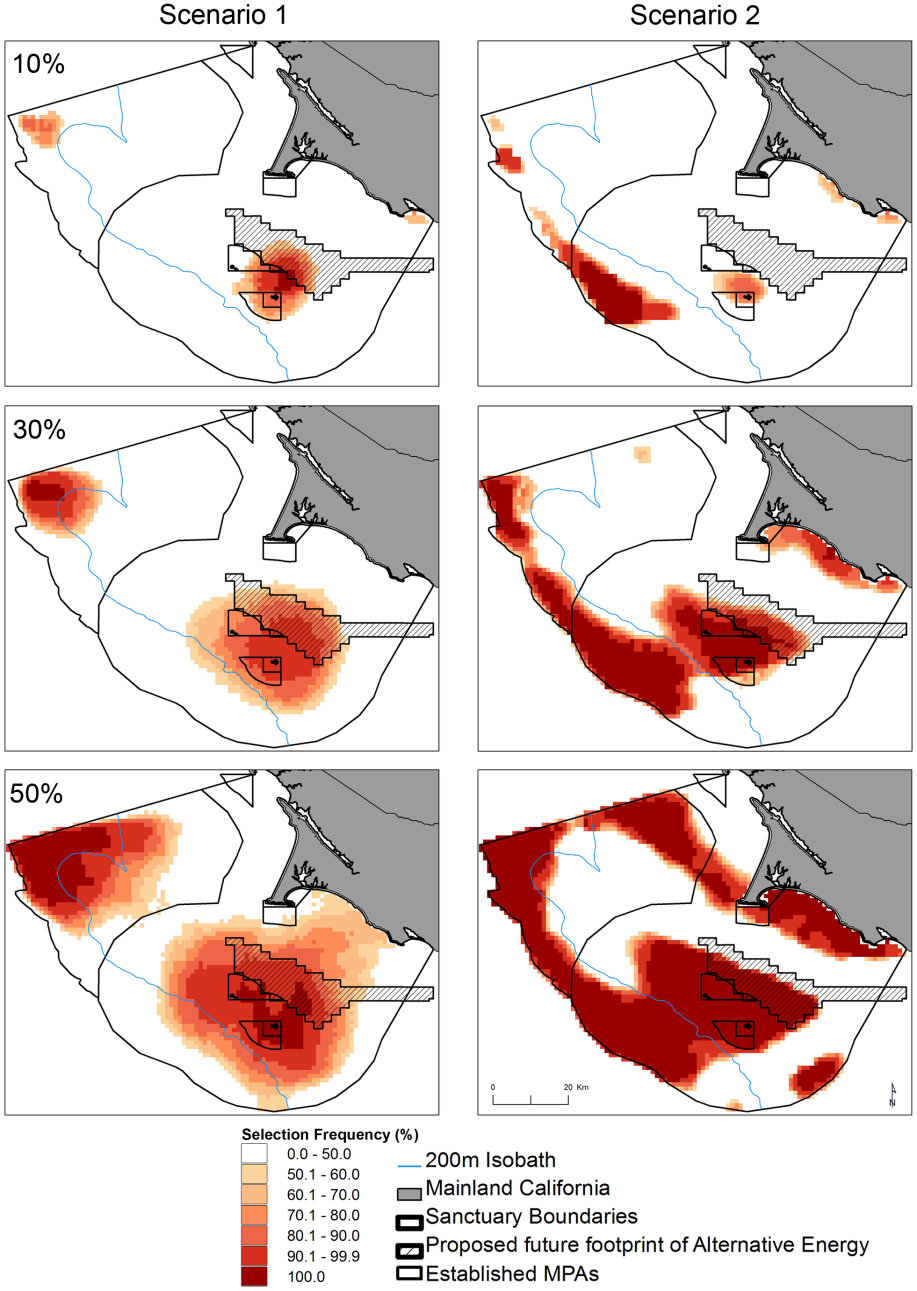
Comparison of Marxan results prioritizing conservation of seabird habitat alone (scenario 1) and with the inclusion of human activities (scenario 2), shown by the cell selection frequency for 10, 30, and 50% conservation targets.

The general human use layer illustrates that the majority of activities occur along the continental shelf east of the 200-m isobath, with higher impacts to seabirds in and adjacent to the shipping lanes (Figure 6). Both scenarios’ solutions satisfied conservation targets by capturing the areas northwest of Cordell Bank, the Farallon Islands, the shelf break, and the nearshore coastline (Figure 7). Differences arose in both the configuration and number of cells required to achieve conservation targets. Scenario 1 selected the areas northwest of Cordell Bank and the waters surrounding the Farallon Islands which our models show to be important multi-species foraging habitat (Figure 5). Scenario 2 also selected these areas, but the inclusion of human uses moved the highest concentrations of selected cells to the low-cost region west of the shelf break and expanded the solutions to include the coastal areas north and south of Point Reyes. In all instances, the best solutions derived from the 100 runs showed that scenario 1 achieved seabird conservation targets using less area than scenario 2 (approximately 50, 75, and 95 km^2^ respectively for the 10, 30 and 50% targets). Visualizing the selection frequency of each cell is a helpful tool Marxan provides to compare the relative importance of certain units over others in finding efficient solutions. We mapped the upper 50% of frequently selected outputs for each scenario’s summed solutions (Figure 7).

## Discussion

For the scale of this study, using shipboard estimates of oceanographic surface data collected simultaneously with species counts likely produced more accurate predictive models than would be possible using only remotely-sensed data. For example, three out of the four species also modeled by Nur et al. [24] were not shown to display significant associations with oceanographic variables at the scale of the CCS. It is not uncommon for large-scale studies to identify relationships with static bathymetric features and basin-wide climate indices when explaining seabird distributions [19], [24]. Such studies show that emergent seabird distribution patterns are scale-dependent and emphasize the critical consideration of scale when approaching management strategies. Our findings fall in line with previous studies but also show that surface oceanography influences habitat selection at local scales. *In situ* data collection captures small variations in surface waters which were important for four out of five of our focal species.

Observed SST and SSS exhibited consistently small ranges of variability when compared to observed SSF. This occurs naturally as phytoplankton bearing chl-a bloom after periods of strong upwelling, resulting in a heterogeneous SSF distribution influenced by water mass type, stratification and the presence of fronts [74]. Using linear regression to fill gaps on cruises lacking SSF data contributed to a smaller predictive variance than may have been otherwise observed if data records were available for all cruises. Even so, SSF data, acting as a proxy for primary productivity, was deemed the best *in situ* data available to include in the modeling exercise to help differentiate productive from non-productive waters. Geostatistical interpolation allowed for rapid and efficient creation of many oceanographic surface layers. While we did not account for the directional influence of currents and water densities on surface property distributions, the overall performance of interpolated surfaces reflected observed patterns and general trends in the surface oceanography of the study region.

Our individual species models helped us identify important foraging habitats utilized by focal species of the Farallon Islands throughout the breeding season. Our five species included two omnivorous, two piscivorous and one zooplanktivorous seabird with differing foraging ecologies. The omnivorous western gulls and common murres foraged closer to the Farallon Islands and over the continental shelf. Western gulls are generalist foragers and their distribution models demonstrated less sensitivity to ocean variability than the other focal species. The importance of nearshore foraging habitats for common murres was evidenced by the necessity to include distance to the mainland for this species alone as a covariate in the model which resulted in improved distribution maps that better reflected observed patterns in habitat use. The piscivores differed by their habitat choices with Brandt’s cormorants traveling to shallower nearshore waters to obtain prey in or near the benthos, and rhinoceros auklets foraging along the 200-m isobath and over Cordell Bank. The zooplanktivorous Cassin’s auklet foraged in the vicinity of Cordell Bank and the Farallon Islands where bathymetric features gather krill [34]. Overall, this component of the analysis offers an understanding of foraging distribution patterns at a scale useful for Sanctuary level management.

Cross-validation results showed that models captured spatial trends and identified high-use areas within the survey region. The maximum distance to the colony on SEFI modeled in the prediction matrix was 90,124-m, almost 25,000-m beyond survey coverage. Extrapolations of species distribution models outside of the survey region are likely subject to increased uncertainty and should be interpreted with caution [48], [75]. For example, models for western gulls and Brandt’s cormorants predicted mirrored distributions west of the 200-m isobath where no transect data exists. Models for Cassin’s auklets and Brandt’s cormorants exaggerated trends in abundance to the edge of the Sanctuaries when extrapolated beyond survey coverage. However, these species-specific artifacts did not persist once incorporated into the composite multi-species foraging habitat map.

Multi-species examinations of highly selected foraging areas are an essential requirement for conservation planning to aid managers in the delineation of candidate sites for marine protection [76]. The core high-use areas identified around Cordell Bank and near the Farallon Islands, as well as the shelf break should be prioritized when considering conservation strategies for seabird management in Sanctuary waters. It should be noted that this study focused primarily on breeding season data, the time of year when seabirds are most abundant and spatially constrained to the colonies. This study also only considered five of forty-three seabird species observed in Sanctuary waters on ACCESS cruises. Studies that examine winter habitat use and include both resident and migratory seabirds will complement these findings for year-round management schemes.

A thorough spatial understanding of human activities and their impacts is an important contributing factor to comprehensive management. Of the activities included in this study, the highest concentrations occurred in GFNMS along the continental shelf due to shipping, fishing and wildlife viewing. Military activities and shipping lanes made up the predominant human activities occurring in CBNMS. We enhanced the implications of both model results and human activity layers by moving them beyond basic visualization and integrating them into a spatial prioritization exercise. While the general human use index generated in this study is useful to demonstrate the utility of these types of exercises for management decisions, more sophisticated human use layers need to be developed for federal waters to improve the performance of such applications. More specifically, data on the frequency and timing of human activities, effort, and a finer understanding of their offshore distributions will be critical to marine spatial planning and zoning within the Sanctuaries.

We used Marxan to illustrate how improved knowledge of species habitat use can be applied to conservation within the Sanctuaries. Our scenarios reflected two approaches that met different management objectives: seabird conservation alone (scenario 1), and seabird conservation while considering the impact of human activities (scenario 2). The latter approach has been applied in state waters adjacent to the Sanctuaries and encompasses the principles of marine spatial planning [36], [77]. Incorporating human uses shifted cell selection towards the offshore region west of the 200-m isobath. There are several management implications we can glean from this analysis: 1) incorporating human activity constrains the high-use foraging areas near the Farallon Islands and Cordell Bank and shifts priority conservation areas to the marginal seabird habitat occurring further offshore, west of the 200-m isobath where human activities occur less frequently. This is relevant to conservation efforts, as seabirds will continue to forage in areas with abundant prey regardless of human activities. Therefore placing seabird conservation areas offshore to avoid conflict with ocean users will be less effective than prioritizing areas identified as high-use; 2) Should conservation areas be established outside of highly-selected foraging habitat, more sub-optimal coastal foraging areas would have to be protected to meet conservation targets. This would be potentially more costly to manage and the ability of these areas to meet the dietary needs and energetic demands of breeding seabirds is unknown; 3) According to the parameters set in this study, there is significant spatial overlap between scenario results demonstrating there is compatibility between seabird conservation areas and human activities currently allowed within Sanctuary waters.

We present results in relation to the footprints of existing state MPAs and potential future alternative energy sites identified by the California Ocean Uses Atlas. As a basic overlay, current MPAs near the Farallon Islands perform better than inshore MPAs at capturing highly selected areas under both scenarios, particularly for 30 and 50% targets. Expansion of these MPAs or protection of contiguous areas could have significant conservation benefits for seabirds. However, the novel contribution of this analysis is to inform ongoing marine spatial planning efforts aimed to regulate emerging ocean uses as well. Alternative offshore energy development is the most notable new management concern for the Sanctuaries. Past proposals and studies outlined areas to deploy wind and wave farm structures on the shelf, east of the Farallon Islands [78]. The effect of these technologies on natural and living resources is unknown, yet actively researched [79]. Our study shows there is considerable spatial overlap between potential seabird conservation areas identified in both scenarios and the proposed alternative energy development footprint (Figure 7). Documented risks to seabirds from wind turbines include collision mortality, habitat loss, displacement and disturbance [80], suggesting this proposed siting area is incompatible with seabird conservation efforts and further analysis should be conducted following the results of this study.

Our ultimate goal was to offer a pragmatic example of how long-term monitoring data can be applied to conservation planning at a localized scale. Our approach investigated the spatio-temporal relationships between wildlife and environmental characteristics driving habitat use patterns. Using seabirds as an example, we present a template on which to build further species-specific models that in turn could be incorporated into an ecosystem-level analysis including main forage species, marine mammal and migratory seabirds. As knowledge improves regarding the use of resources in Sanctuary waters by both wildlife and humans, more sophisticated studies can supplement conservation decisions and minimize threats to marine species and living resources. This approach can be applied elsewhere to support marine spatial planning efforts for current and future conservation objectives.
